# Serum Protein N-Glycosylation Signatures of Neuroblastoma

**DOI:** 10.3389/fonc.2021.603417

**Published:** 2021-03-16

**Authors:** Wenjun Qin, Hao Pei, Xiaobing Li, Jia Li, Xuelian Yao, Rufang Zhang

**Affiliations:** ^1^ Department of Pediatric Cardiothoracic Surgery, Shanghai Children’s Hospital, Shanghai Jiao Tong University, Shanghai, China; ^2^ Department of Anesthesiology, Children’s Hospital of Fudan University, Shanghai, China

**Keywords:** neuroblastoma, N-glycosylation, serum N-glycomics, mass spectrometry, disease marker

## Abstract

**Background:**

Neuroblastoma is the most common extracranial childhood solid tumor which accounts for 10% of the malignancies and 15% of the cancer fatalities in children. N-glycosylation is one of the most frequent post-translation protein modification playing a vital role in numerous cancers. N-glycosylation changes in neuroblastoma patient serum have not been studied in existing reports. The comprehensive analyses of serum N-glycomics in neuroblastoma can provide useful information of potential disease biomarkers and new insights of the pathophysiology in neuroblastoma.

**Methods:**

The total serum protein N-glycosylation was analyzed in 33 neuroblastoma patients and 40 age- and sex-matched non-malignant controls. N-glycans were enzymatically released, derivatized to discriminate linkage-specific sialic acid, purified by HILIC-SPE, and identified by MALDI-TOF-MS. Peak areas were acquired by the software of MALDI-MS sample acquisition, processed and analyzed by the software of Progenesis MALDI.

**Results:**

Three glyco-subclasses and six individual N-glycans were significantly changed in neuroblastoma patients compared with controls. The decreased levels of high mannose N-glycans, hybrid N-glycans, and increased levels of α2,3-sialylated N-glycans, multi-branched sialylated N-glycans were observed in neuroblastoma patients. what is more, a glycan panel combining those six individual N-glycans showed a strong discrimination performance, with an AUC value of 0.8477.

**Conclusions:**

This study provides new insights into N-glycosylation characteristics in neuroblastoma patient serum. The analyses of total serum protein N-glycosylation could discriminate neuroblastoma patients from non-malignant controls. The alterations of the N-glycomics may play a suggestive role for neuroblastoma diagnosis and advance our understanding of the pathophysiology in neuroblastoma.

## Introduction

Neuroblastoma (NB) is the most common type of extracranial solid tumor in children, accounting for about 8–10% of pediatric malignancies and for 15% of malignant neoplasm deaths in children (1, 2). It derives from the neural crest cells of the sympathetic system, which can arise along the sympathetic ganglion chain from the neck to the pelvis. Approximately 64% of neuroblastomas are diagnosed in the abdomen, 14% are diagnosed in the posterior mediastinum, and the remainders are diagnosed in the neck, pelvis and other locations (3). A key characteristic of neuroblastomas is their extreme heterogeneity, generating different clinical presentations (4). A small number of them can spontaneously differentiate and regress with a favorable prognosis even without any therapies, but most of them are highly undifferentiated displaying very aggressive behaviors, refractory to current therapies, and with very poor outcomes (5).

With the development of the risk classification system introduced by International Neuroblastoma Risk Group (INRG), patients are divided into different risk categories according to their clinical markers such as age, tumor stage, and histology as well as genetic markers such as *MYCN* amplification and arm-level alterations of chromosomes (6). In this way, risk-assigned therapies have been delivered to NB patients, which have improved the prognosis to some extent (7). Nevertheless, the diagnoses of some patients are still delayed due to the occult locations of occurrence and the lack of specific clinical symptoms. Overall, more than 50% of new diagnosed neuroblastomas are metastatic (8). Despite aggressive multi-modal treatments such as resection, intensive chemotherapy, radiation, immunotherapy and 13-cis-retinoic acid are applied, the long-term survival of the high risk patients with metastasis remains below 50% (9, 10). Currently, the standardized criteria for diagnosis of neuroblastomas include the analysis of urinary catecholamine metabolites, imaging procedures and histopathology (11). However, in pediatric patients, it has difficulty in collecting 24-h urine samples. Imaging examination is greatly affected by the location of lesions and the size of tumors. Although histopathology is considered as the gold standard for the diagnosis of NB, which is obtained through invasive surgical procedures and in some cases may not be accessible (9). Peripheral blood based tests are minimally invasive and the risk to patients is negligible. They are easily obtainable and can be repeated at shorter intervals with a higher application value, even in pediatric patients (12). Therefore, incorporating some new serology biomarkers as one part of the diagnostic and prognostic criteria will benefit the patients from receiving appropriate treatments in time.

Glycosylation is one of the most common post-translation protein modification which plays a vital role in numerous biological functions such as protein folding, cell signaling, cell adhesion and immune response (13–15). The levels of expression of N-glycans affect multiple physiological and pathological processes. Aberrant glycosylation has been reported to be associated with many mammalian diseases including immunological disorders (16), inflammations (17), cardiovascular diseases (18) and different type of malignancies (19). Thus, detecting and monitoring N-glycomic differences including the information of structures and the abundance of N-glycans between healthy and diseased individuals is of great interest and importance for advancing our understanding of the diseases. The mass spectrometry (MS) based strategies have been widely employed (20, 21). In neuroblastoma, disialoganglioside (GD2) has been reported as a potential molecular marker for detection and a target for immunotherapy in high-risk NB patients (22). It was reported that reduced N-glycosylation of intercellular adhesion molecule-2 (ICAM-2) attenuated the ability to suppress metastasis of NB cells (23). The inhibition of N-glycosylation of anaplastic lymphoma kinase (ALK) protein which is significantly up-regulated in advanced and metastatic NB affects its phosphorylation and disrupts downstream pro-survival signaling (24). Additionally, a systematic comparison of N-linked glycomic variations between different neuroblastoma cell lines revealed that less galactosylated and more sialylated N-glycan structures were found in *MYCN*-amplified cell lines compared with *MYCN*-nonamplified cell lines (25). And in our previous study, we revealed the deceases of serum IgG galactosylation in neuroblastoma, and this distribution may play a suggestive role for neuroblastoma diagnosis (26). Therefore, analyzing the levels of expression of N-glycans in easily accessible body fluids such as serum and plasm may provide potential biomarkers and facilitate our understanding of the mechanisms and progression of NB. However, to our knowledge, there has no study about a comprehensive N-glycomic profiling of NB patient serum.

In this study, we conducted a comprehensive analysis of the total serum protein N-glycomics purified from only microliter volumes of serum samples derived from NB patients and age- and sex-matched non-malignant controls using matrix-assisted laser desorption/ionization time-of-fight mass spectrometry (MALDI-TOF-MS) in order to identify neuroblastoma-related N-glycan alterations which can provide insights into the biomarkers and pathophysiology of NB. In total, seventy-eight N-glycans were identified, from which 60 N-glycans and eight glyco-subclasses were quantified. Three glyco-subclasses (*p* < 0.0063, after Bonferroni correction) and six N-glycans (*p* < 0.00083, after Bonferroni correction) showed significant alternations. A combination of these six individual glycans could achieve a strong discrimination performance. What is more, the detailed summary of serum N-glycomics in NB can provide useful information of potential disease biomarkers and new insights of the pathophysiology in NB.

## Materials and Methods

### Study Population and Sample Collection

Serum samples were collected from 33 patients diagnosed with neuroblastic tumor consisted of 22 neuroblastoma (NB) cases and 11 ganglioneuroblastoma (GNB) cases which were classified according to the International Neuroblastoma Pathology Classification (27). In addition, 40 serum samples from age- and sex-matched non-malignant individuals were enrolled as controls.

The venous blood samples were obtained preoperatively during the morning fasting state. After clotting 30 min at ambient temperature, the tubes were centrifuged at 2,000 × g for 10 min. The serum samples were aliquoted and stored at −80°C until analyses. The approvals of this study were obtained from the Institutional Review Board of the Children’s Hospital of Fudan University, China [(2019)063] and Shanghai Children’s Hospital, Shanghai Jiao Tong University, China (2020RY014), and informed consents from all participants were acquired.

### N-Glycans Release

Five microliters of serum were denatured at 60°C for 10 min by adding 2 µL of denaturing solution (2% sodium dodecyl sulfate) (Merck, Germany). When it cooling to room temperature, 2 µL of glycobuffer (4% Nonidet P-40, 5×PBS, PH 7.5) (New England Biolabs, USA) and 1 μL peptide-N-glycosidase F (New England Biolabs, USA) were added to the mixture and incubated at 37°C overnight.

### N-Glycans Derivatization

The released N-glycans were subsequently derivatized with ethanol in order to discriminate the ethyl-esterification of 2,6- linked sialic acids and lactonization of 2,3-linked sialic acids as described previously (20). Briefly, the released N-Glycans were added to ethanol (Merck, Germany) which contains 1-ethyl-3-(3-(dimethylamino) propyl)-carbodiimide (EDC) hydrochloride (Fluorochem, UK) and 1-hydroxybenzotriazole monohydrate (HOBt) (Sigma-Aldrich, Germany), each component at a concentration of 0.25 M. The samples were incubated at 37°C for 1h. Acetonitrile (ACN) (Merck, Germany) was added and all samples were further incubated at − 20°C for 15 min to precipitate the protein as reported previously.

### HILIC Solid-Phase Extraction

The derivatized N-Glycans were subsequently enriched using cotton hydrophilic interaction liquid chromatography (HILIC) solid phase extraction (SPE) tips as described previously (20, 28). Briefly, twenty microliter pipet tips (Rainin Instrument, USA) were packed with 3mm cotton thread. The cotton tips were pre-conditioned and equilibrated with 3×20 μL of Milli-Q water and 3×20 μL of 85% ACN. The samples were loaded on the cotton by pipetting more than 20 times. Then, the tips were washed with 3×20 μL of 85% ACN containing 1% trifluoroacetic acid (Sigma-Aldric, Germany) and 3×20 μL of 85% ACN. Finally, the N-Glycans were eluted into the collection plate using 10 μL of Milli-Q water.

### MALDI-QIT-TOF MS Analysis

Before MALDI MS analysis, TOFMix (LaserBio Laboratories, France) containing an eight-peptide calibration standard was employed to calibrate the MS. One microliter of glycan sample was spotted on a standard MALDI plate and allowed to dry in air. Then, 1 μL super-DHB (Sigma-Aldrich, Germany) (5 mg/mL) 1 mM NaOH (Sigma-Aldrich, Germany) in 50% ACN was added onto the plate and allowed to dry by air. To uniform the spot surface, 0.2 μL ethanol was added to recrystallize matrix crystals. Every sample was spotted in triplicate. The samples were analysis by AXIMA Resonance MALDI-QIT-TOF MS (Shimadzu Corp. JP) equipped with a 337 nm nitrogen laser in reflector positive ionization mode. Two laser shots were set to generate a profile, and 200 profiles were accumulated from different points of laser irradiation into one file for each spectrum. All the spots were detected with two measurements for the quantitation for the range of expected N-glycans. In one measurement, the mass range was set at Mid 850 + with a lower laser power 110 V for lower m/z ions (approximately m/z 1,000 to 3,000). In another measurement, the mass range was set at High 2,000 + with a higher laser power 125 V for higher m/z ions (approximately m/z 2,000 to 4,000). The glycan compositions were assigned according to previous literatures (20, 29) and known biosynthetic pathways. The GlycoWorkbench software was used for the annotation of MS spectra.

### Data Processing and Statistical Analysis

The MALDI MS data were acquired and exported as ASCII files using Launchpad software (Shimadzu Biotech, Japan). The ASCII files were pre-processed, normalized and extracted using the software of Progenesis MALDI, and then transferred to Microsoft Excel as text files. The threshold of Progenesis MALDI was set at 1,000, thus the signals with low signal to noise ratio were removed. For the accurate analyses, only N-glycan signals that were present with S/N (signal to noise) more than 3 in the spectra were included for further analyses. The relative area of each N-glycan peak was calculated by setting the sum of normalized peak areas of each sample to one.

As each serum sample was spotted in triplicate, there normalized data for each serum sample were averaged before statistical analysis. Only N-glycans with the coefficient of variations less than 25% according to the quantitation results were included in the statistical analyses. For statistical analysis, the averaged data were performed with GraphPad Prism 7 and SPSS (version 16.0) to identify possible alterations in the levels of N-glycans in different groups. The statistically significant difference was evaluated by performing t-test with Bonferroni correction. The data were further processed by the receiver-operator-characteristics (ROC) test to assess the specificity, sensitivity, and accuracy of the potential diagnostic variable. Then, the values of area-under-the-curve (AUC) with 95% confidence intervals (95% CI) was assigned. If the AUC value was greater than 0.9, the tests were considered “highly accurate,” while values between 0.8 and 0.9 were deemed “accurate”. When the AUC value was between 0.7 and 0.8, the test was concluded to be “moderately accurate”. An “uninformative” test resulted in an AUC value that was between 0.5 and 0.7. To test the reproducibility of this methodology, the workflow was repeated three times for one sample, the average coefficient of variation (CV) of all the glycans for quantification was 11.42%.

## Results

### Clinical Samples

In this study, a subset of 33 serum samples was examined from patients with neuroblastoma (NB) (N = 22) and ganglioneuroblastoma (GNB) (N = 11) which were classified according to the International Neuroblastoma Pathology Classification (27). They ranged in age from 2 months to 130 months (mean, 52.13 months). The primary sites were retroperitoneum in 18 patients, adrenal gland in 6 patients, and mediastinum in 9 patients. Eight of these patients were diagnosed at stage I and II, twenty-four of them were diagnosed at advanced stage III and IV, and one of them was diagnosed at stage IVs according to the International Neuroblastoma Staging System (INSS) (30). In addition, 40 age- and sex-matched non-malignant individuals were enrolled as controls ageing from 12 months to 144 months (mean, 66.35 months) including 9 healthy volunteers, 16 fracture cases, 9 hernia cases, 4 phimosis cases and 2 hydrocele cases. The clinicopathological and baseline demographic characteristics including age, sex, INSS stage, and histological types were listed in **Table 1**.

**Table 1:** Characteristics of neuroblastoma patients and non-malignant controls.

Characteristic	Neuroblastoma patients		Non-malignant controls		p-value
	No.	%	No.	%	
**Age (months)**					>0.05
** mean ± SD**	52.13 ± 30.24		66.35 ± 33.99		
** Median**	48.5		60		
** Range**	2–130		12–144		
**Gender**					>0.05
** Male**	22	66.67	28	70.00	
** Female**	11	33.33	12	30.00	
**INSS stage**					
** I,II**	8	24.24	–	–	
** III,IV**	24	72.72	–	–	
** IVs**	1	3.03	–	–	
**Histological type**					
** NB**	22	66.67	–	–	
** GNB**	11	33.33	–	–	
**Type**			–	–	
** Healthy cases**	–	–	9	22.50	
** Fracture cases**	–	–	16	40.00	
** Hernia cases**	–	–	9	22.50	
** Phimosis cases**	–	–	4	10.00	
** Hydrocele cases**	–	–	2	5.00	

INSS, International Neuroblastoma Staging System; NB, neuroblastoma; GNB, ganglioneuroblastoma.

### Serum N-Glycan Profiles

**Figure 1:**
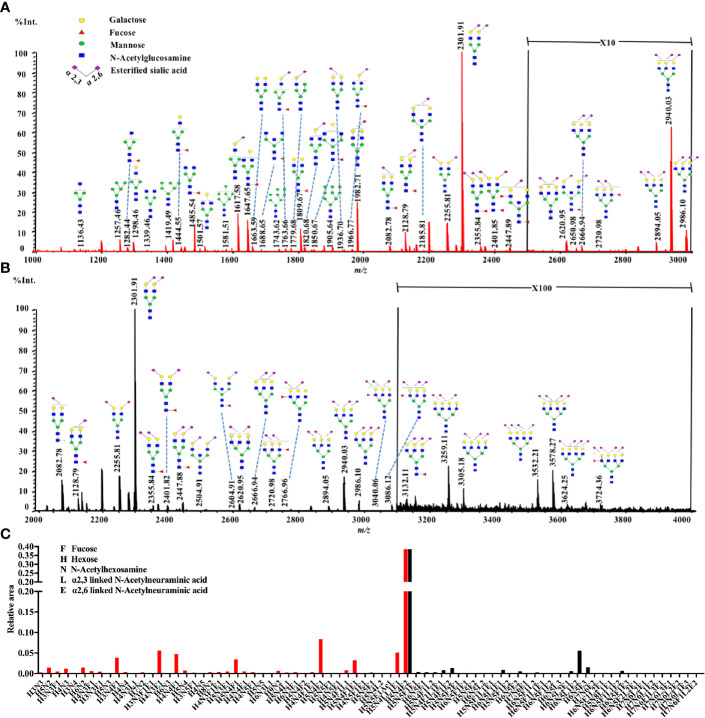
A typical MALDI-QIT-TOF MS spectrum of serum N-glycan profiles from a serum sample. **(A)** The representative spectrum of low-mass measurement (approximately m/z 1,000 to 3,000). **(B)** The representative spectrum of high-mass measurement (approximately m/z 2,000 to 4,000). **(C)** Combination of the low mass (red) and high mass (black) area integration originating from a single spot. The relative areas were normalized to the peak at m/z 2301.91, reflecting the N-glycan composition [H5N4E2 + Na]^+^.

The total serum N-glycans were enzymatically released from total serum proteins by PNGase F, derivatized by ethanol with EDC and HOBt which can discriminate linkage-specific sialic acids, purified by HILIC-SPE, and identified by MALDI-TOF-MS. All the spots were detected with two measurements for the quantitation for the range of expected N-glycans according to the previous study (31). A total of 438 mass spectra were obtained from low-mass and high-mass measurements of MALDI spots. The representative spectra of low-mass (**Figure 1A**) and high-mass (**Figure 1B**) measurements from one non-malignant control are shown in **Figure 1**. The relative abundances were normalized to the peak m/z 2301.91 (H5N4E2). In this way, the low-mass and high-mass spectra of every spot were combined for the quantification of relative peak area (**Figure 1C**). Ultimately, a total of 78 N-glycans with S/N (signal to noise) more than three could be detected and identified based on previous literatures, ranging from H3N3 (m/z 1136.43) to H7N6F1L2E2 (m/z 3724.40) (**Supplemental Table 1**).

### Analysis of Different N-Glycan Subclasses in NB and Controls

Of the 78 N-glycans detected in NB and control serum samples, only 60 N-glycans (labeled with red in **Supplemental Table 1**) with the CVs less than 25% according to the quantitation results were included in the statistical analyses. To investigate the differences of serum protein N-glycosylation between NB patients and non-malignant controls, initially, the 60 N-glycan structures were classified into eight specific subclasses based on their characteristic structural features. The glyco-subclasses included terminal-galactosylated N-glycans, fucosylated N-glycans, high mannose N-glycans, hybrid N-glycans, α2,3-sialylated N-glycans, α2,6-sialylated N-glycans, bisecting N-glycans and multi-branched sialylated N-glycans. The eight specific subclasses were calculated according to the formulas shown in the **Supplemental Table 2**.

**Figure 2:**
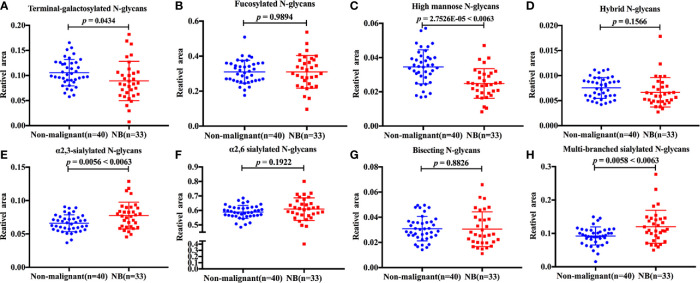
The relative abundance of eight glyco-subclasses in NB patients and non-malignant controls. The N-glycans were grouped according to their structural characteristics. **(A)** Terminal-galactosylated N-glycans; **(B)** Fucosylated N-glycans; **(C)** High mannose N-glycans; **(D)** Hybrid N-glycans; **(E)** α2,3-sialylated N-glycans; **(F)** α2,6-sialylated N-glycans; **(G)** Bisecting N-glycans; **(H)** Multi-branched sialylated N-glycans. The *p* value < 0.05/8 = 0.0063 was considered statistically significant after Bonferroni correction.

The statistical analyses were performed following the summation of the normalized relative abundance of every N-glycan in each glyco-subclass. The *p* value < 0.05/8 = 0.0063 was considered statistically significant after Bonferroni correction. The comparisons of these eight glyco-subclasses between NB and controls are displayed in **Figure 2**. Three of these glyco-subclasses showed significant alterations in NB patients including high mannose N-glycans, α2,3-sialylated N-glycans, and multi-branched sialylated N-glycans. A decrease in the levels of high mannose N-glycans (**Figure 2C**) appeared in NB patients. In contrast, α2,3-sialylated N-glycans (**Figure 2E**) and multi-branched sialylated N-glycans (**Figure 2H**) showed significantly increased levels in NB patients compared with non-malignant controls. No significant differences in terminal-galactosylated N-glycans, fucosylated N-glycans, hybrid N-glycans, α2,6-sialylated N-glycans, and bisecting N-glycans were observed between these two groups (**Figures 2A, B, D, F, G**).

**Figure 3:**
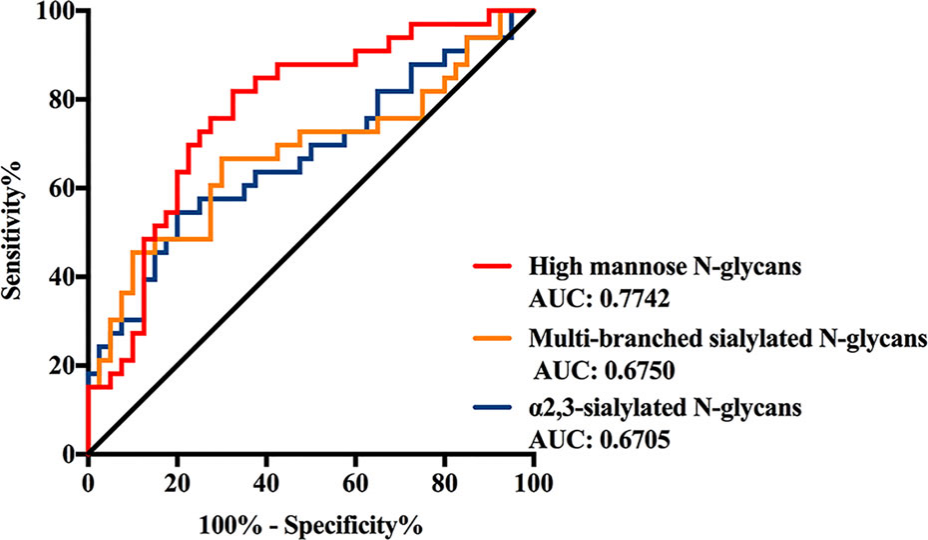
Receiver operating characteristic (ROC) curve analyses for the three significantly changed glyco-subclasses. The ROC was employed to evaluate the discrimination efficiency of these glyco-subclasses including High mannose N-glycans, α2,3-sialylated N-glycans, Multi-branched sialylated N-glycans. Their AUC values were 0.7742, 0.6750, 0.6705 respectively.

The discrimination efficiency of these three glyco-subclasses with significant changes was evaluated through receiver operating characteristic (ROC) analyses (**Figure 3**). The glyco-subclass of α2,3-sialylated N-glycans presented an AUC of 0.6705 (95% CI: 0.5433 to 0.7976) and the glyco-subclass of multi-branched sialylated N-glycans showed an AUC of 0.6750 (95% CI: 0.5461 to 0.8039), suggesting an uninformative discrimination of NB. While the AUC value of high mannose N-glycans was 0.7742 (95% CI: 0.6652 to 0.8833), which demonstrated the potential utility for NB diagnosis.

### Identification of Specific N-Glycan Structure Differences Between NB and Controls

A more detailed examination was performed to investigated the differences of those 60 N-glycans between NB patients and non-malignant controls. The glycoforms, relative peak area, p-value and AUC were listed in **Supplemental Table 3**. A total of 23 N-glycans were observed with alterations in their expression levels between these two groups (p < 0.05). On account of 60 N-glycans were performed for statistical analysis in this part, the p value < 0.05/60 = 0.00083 was considered statistically significant after Bonferroni correction.

**Figure 4:**
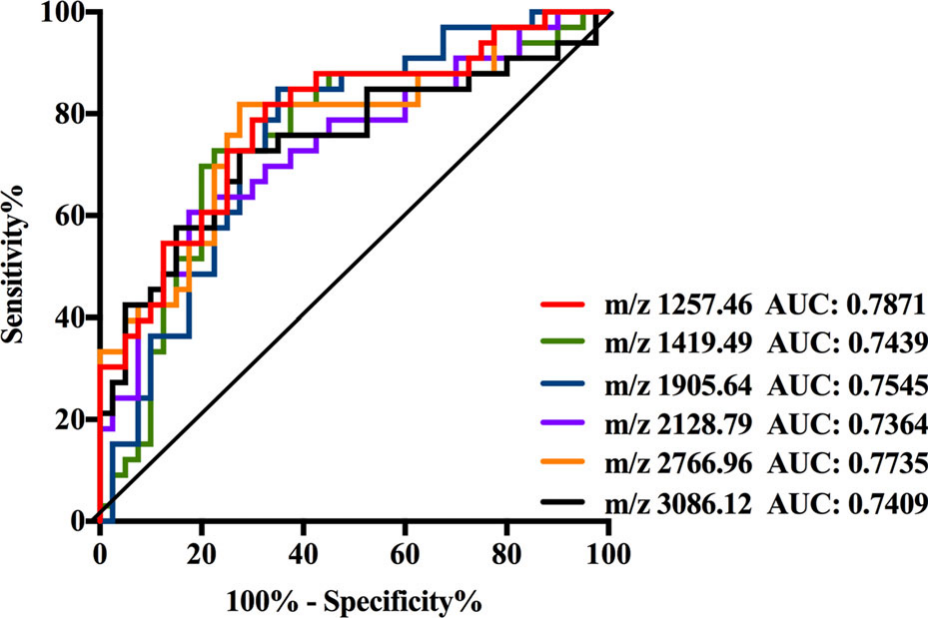
Receiver operating characteristic (ROC) curve analyses for the six significantly changed individual N-glycan. The ROC was employed to evaluate the discrimination efficiency of these N-glycans including: m/z1257.46, 1419.49, 1905.64, 2128.79, 2766.96, and 3086.12. Their AUC values were 0.7871, 0.7439, 0.7545, 0.7364, 0.7735, and 0.7409 respectively.

As shown in **Supplemental Table 3**, the data of six N-glycans generated *p*-values less than 0.00083 including three high mannose structures (m/z 1257.46, 1419.49, and 1905.64), one fucosylated sialylated biantennary structure (m/z 2128.79), and two fucosylated tri-branched structures with various levels of sialylation (m/z 2766.96 and 3086.12). Among these glycans, those four N-glycans observed at m/z 1257.46, 1419.49, 1905.64, and 2128.79 significantly decreased in NB patients resulted in AUC values of 0.7871, 0.7439, 0.7545, and 0.7364. While the expression levels of those two fucosylated tri-branched structures (m/z 2766.96 and 3086.12) were elevated by more than two-fold in NB patients compared with non-malignant controls (m/z 2766.96: NB vs. Control, 0.0045 to 0.0022; m/z 3086.12: NB vs. Control, 0.0241 to 0.0110), presenting AUC values of 0.7735 and 0.7409. The AUC values of all the six glycans were higher than 0.70 (**Figure 4**) with moderate accurateness, indicating that these glycans have the potential to detect the presence of NB.

### Construction and Evaluation of a Diagnostic Model Based on N-Glycan Markers to Discriminate NB and Controls

**Figure 5:**
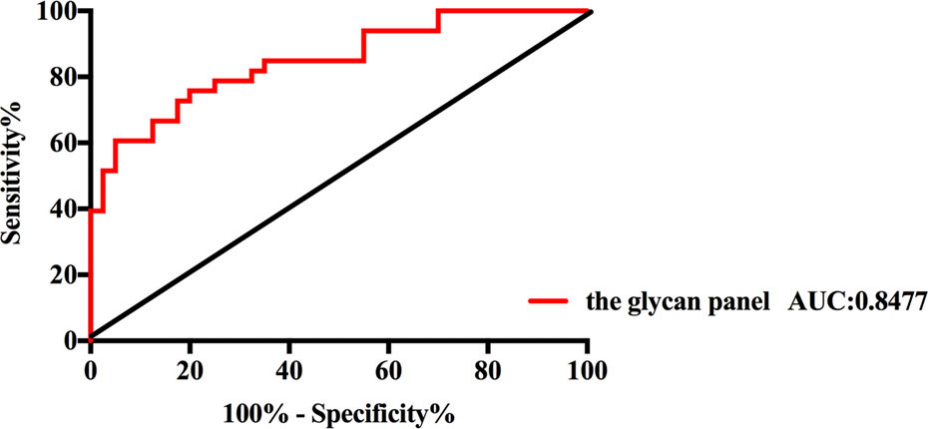
Receiver operating characteristic (ROC) curve analysis for the glycan panel. The ROC was employed to evaluate the discrimination efficiency of the glycan panel. Its AUC value was 0.8477.

All of those three glyco-subclasses and six glycans which were identified as being significantly changed in NB patients showed a moderately accurateness in discriminating NB from controls. Thus, a more accurate prediction model should be established. We integrated different glycan panels to better discriminate NB patients from controls based on the logistic regression analysis. Indeed, we found that a glycan panel combing these six glycans played a better discrimination performance for NB patients and controls. The AUC values of these three glyco-subclasses, six glycans and the glycan panel were compared so as to compare their sensitivities and specificities of distinguishing NB patients from controls (**Table 2**). For the glyco-subclasses and individual glycans, the AUC values ranged from 0.6705 to 0.7871, and the AUC score of the glycan m/z 1257.46 was the largest, with a sensitivity of 81.82% and specificity of 67.50%. Significantly, the AUC score of the combined glycan panel was 0.8477 (**Figure 5**), which higher than 0.80, with a specificity of 95.00% and specificity of 60.61%, indicating its strong discrimination performance. Thus, it may play a suggestive role for neuroblastoma diagnosis.

**Table 2:** List of the three glyco-subclasses, six specific N-glycans and the glycan panel that were evaluated to be specific for neuroblastoma patients compared with non-malignant controls by Receiver Operating Characteristic (ROC) curve analysis.

Glycan	AUC	St. Error	95% CI	*P* value	Sensitivity	Specificity
α2,3-sialylated N-glycans	0.6705	0.0649	0.5433–0.7976	0.0126	54.55%	80.00%
multi-branched sialylated N-glycans	0.6750	0.0658	0.5461–0.8039	0.0105	66.67%	70.00%
high mannose N-glycans	0.7742	0.0556	0.6652–0.8833	<0.0001	81.82%	67.50%
m/z 1257.46	0.7871	0.0541	0.6812–0.8931	<0.0001	81.82%	67.50%
m/z 1419.49	0.7439	0.0606	0.6251–0.8628	0.0004	72.73%	77.50%
m/z 1905.64	0.7545	0.0571	0.6426–0.8664	0.0002	84.85%	65.00%
m/z 2128.79	0.7364	0.0595	0.6198–0.8529	0.0005	60.61%	82.50%
m/z 2766.96	0.7735	0.0564	0.6630–0.8840	<0.0001	81.82%	72.50%
m/z 3086.12	0.7409	0.0610	0.6213–0.8605	0.0004	72.73%	72.50%
the glycan panel	0.8477	0.0453	0.7589–0.9365	<0.0001	60.61%	95.00%

## Discussion

To our knowledge, this is the first attempt to quantitatively evaluate the changes of total serum protein N-glycosylation in NB patients. The alteration in N-glycosylation is a hallmark of cancers (32). Here, we employed a fast, easy and mild esterification method which can discriminate linkage-specific sialic acids with MALDI-TOF MS to reveal the alterations of N-glycosylation. And the quantitation of N-glycans was successfully achieved by using microliter volumes of serum samples. In total, we identified 78 N-glycans in all serum samples. From those, 60 N-glycans and eight glyco-subclasses were statistically analyzed. Our data showed that the expression levels of three glyco-subclasses and six individual N-glycans were significantly different between NB patients and non-malignant controls after Bonferroni correction. What is more, we constructed a glycan penal which could discriminate NB patients from controls with an AUC score of 0.8477.

Although the alterations of high mannose structures have been reported in different cancers (33–35), the possible changes of high mannose structures in NB have not been addressed in existing studies. In our study, the levels of high mannose structures were found significantly decreased in NB patients. High mannose structures are the precursor glycans for hybrid glycans and complex glycans which are more mature types. The synthesis of N-glycans is initiated in the endoplasmic reticulum, where a glycan precursor consisting of three glucoses, nine mannoses, and two N-acetylglucosamines is transferred to the protein followed by the removal of glucoses to form a high mannose glycan. Subsequently, the protein is transferred to the Golgi apparatus, and the high mannose structures can be decorated by different enzymes to synthesize the hybrid and complex type glycans (15). The decrease of the relative abundance of high mannose structures in NB may be caused by the decreased level of the proteins rich in high mannose structures such as alpha-2-macroglobulin, apolipoprotein B-100, immunoglobulin D, immunoglobulin E and immunoglobulin M (36) or the improvement of the synthesis efficiency of complex type glycans.

The aberrant alterations in sialylated N-glycans were also observed in the present study which have been well reported in various cancers including NB cells (25). α2,3-linked sialic acids have been reported to contribute to the biosynthesis of sialyl-Lewis epitopes, which are well-known correlated with malignant progression and poor prognosis in cancers (37–39). While α2,6-linked sialic acids promote the survival of tumor cells by their negative regulation of galectin binding (40, 41). Owing to the different functions of α2,3 and α2,6-linked sialic acids, we employed a derivatization method which could provide linkage-specific sialic acid information. Indeed, we found that only α2,3-linked sialic acids increased in NB patients compared with non-malignant controls. Furthermore, increased amounts of multi-branched sialylated N-glycans were detected in NB. The formation of a sialyl-Lewis epitope requires an antenna fucose and an α2,3-linked sialic acid (42). Fucoses mostly present in the core in di-antennary N-glycans, but in multi-branched N-glycans, fucoses are usually connected to the antennas (43). Thus, the elevation of the levels of both α2,3-sialylated N-glycans and multi-branched sialylated N-glycans might indicate the increase of the sialyl-Lewis epitopes in NB patients. And we further investigated the MS/MS behavior of the multi-branched α2,3-sialylated N-glycan (m/z 3086.12) which significantly increased in NB patients to confirm the glycan structure. As shown in **Supplemental Figure 1**, the ions at m/z 807.0 and m/z 969.0 were observed confirming its sialyl-Lewis epitope.

On account of the insufficient sample size in this study, we were not able to analyze the differences between different histological subtypes and clinical stages. Further studies are still needed to validate the potential of these N-glycans as biomarkers and reveal the role of protein glycosylation in NB pathophysiology.

## Conclusion

In conclusion, we carried out a detailed study of the total serum protein N-glycosylation in NB patients by MALDI-QIT-TOF MS with microliter volumes of serum samples. Significant changes in the expression levels of three glyco-subclasses and six individual N-glycans were identified in NB patients compared with non-malignant controls. The combination of these individual glycans can increase the discrimination accurateness, with an AUC of 0.8477, providing a potential diagnosis biomarker for NB. And this study provides new insights of the pathophysiology in NB. Further studies for validation and into the biochemical mechanisms of the glycomic changes based on our observations are necessary to improve our understanding of NB.

## Data Availability Statement

The original contributions presented in the study are included in the article/**Supplementary Material**. Further inquiries can be directed to the corresponding author.

## Ethics Statement

The studies involving human participants were reviewed and approved by the Institutional Review Board of the Children’s Hospital of Fudan University, China and Shanghai Children’s Hospital, Shanghai Jiao Tong University, China. Written informed consent to participate in this study was provided by the participants’ legal guardian/next of kin.

## Author Contributions

RZ conceived and initiated this study. WQ designed the experiments, interpreted the data, and drafted the manuscript. HP and WQ collected samples and provided clinical information. XL, JL, and XY contributed to the research materials. All authors contributed to the article and approved the submitted version.

## Funding

The authors acknowledge the financial support of faculty start-up grant from the Children’s Hospital of Shanghai, Shanghai Jiao Tong University and the Interdisciplinary (Engineering-Medical) Research Fund of Shanghai Jiao Tong University (YG2021QN120).

## Conflict of Interest

The authors declare that the research was conducted in the absence of any commercial or financial relationships that could be construed as a potential conflict of interest.
