# Supplementary material for: Serum Protein N-Glycosylation Signatures of Neuroblastoma
Source: Front Oncol. 2021 Mar 16;11:603417. doi: 10.3389/fonc.2021.603417 (PMC8008057; doi:10.3389/fonc.2021.603417)
Supplement: Supplementary file 1 [file DataSheet_1.docx]

**Supplementary** **materials**

**Serum Protein N-glycosylation Signatures of Neuroblastoma**

Wenjun Qin ^1#^, Hao Pei^2#^, Xiaobing Li^1^, Jia Li^1^, Xuelian Yao^1^, Rufang, Zhang^1*^

**Affiliations**

^1^ Department of Pediatric Cardiothoracic Surgery, Shanghai Children’s Hospital, Shanghai Jiao Tong University, Shanghai 200062, China.

^2^ Department of Anesthesiology, Children's Hospital of Fudan University, Shanghai, 201102, China.

^#^ These authors contributed equally to this work.

^*^Corresponding author:

Rufang, Zhang: E-mail: zhangrufangshch@163.com; Tel: +86-021-52976201.

**Supplemental Table 1.** The list of N-glycans identified in human serum.

| **No** | **Putative structure** | **Composition** | **MW(M + Na)^+^** | |
| --- | --- | --- | --- | --- |
|  |  |  | **Calculated** | **Observed** |
| 1 | 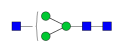 | H3N3 | 1136.40 | 1136.43 |
| 2 | 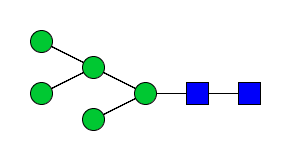 | H5N2 | 1257.42 | 1257.46 |
| 3 | 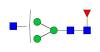 | H3N3F1 | 1282.45 | 1282.44 |
| 4 | 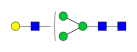 | H4N3 | 1298.45 | 1298.46 |
| 5 | 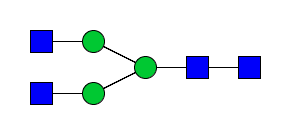 | H3N4 | 1339.48 | 1339.46 |
| 6 | 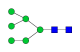 | H6N2 | 1419.48 | 1419.49 |
| 7 | 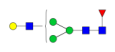 | H4N3F1 | 1444.51 | 1444.55 |
| 8 |  | H3N3E1 | 1455.52 | 1455.56 |
| 9 | 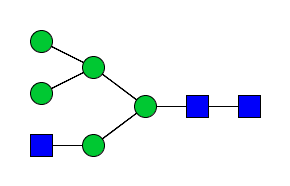 | H5N3 | 1460.50 | 1460.48 |
| 10 | 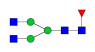 | H3N4F1 | 1485.53 | 1485.54 |
| 11 | 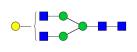 | H4N4 | 1501.53 | 1501.57 |
| 12 |  | H4N3L1 | 1571.53 | 1571.57 |
| 13 | 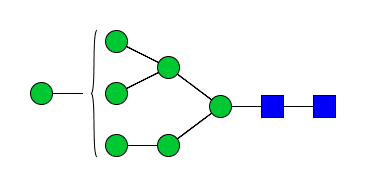 | H7N2 | 1581.53 | 1581.51 |
| 14 | 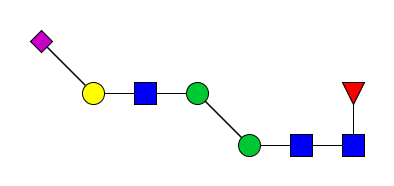 | H3N3F1E1 | 1601.58 | 1601.56 |
| 15 | 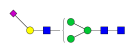 | H4N3E1 | 1617.58 | 1617.58 |
| 16 | 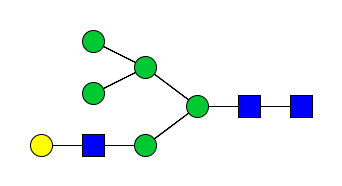 | H6N3 | 1622.55 | 1622.59 |
| 17 | 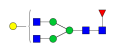 | H4N4F1 | 1647.59 | 1647.65 |
| 18 | 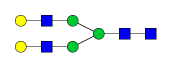 | H5N4 | 1663.58 | 1663.59 |
| 19 |  | H3N5F1 | 1688.61 | 1688.65 |
| 20 |  | H4N5 | 1704.61 | 1704.59 |
| 21 |  | H8N2 | 1743.58 | 1743.62 |
| 22 | 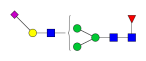 | H4N3F1E1 | 1763.63 | 1763.66 |
| 23 |  | H5N3E1 | 1779.63 | 1779.68 |
| 24 | 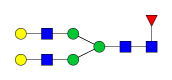 | H5N4F1 | 1809.64 | 1809.67 |
| 25 | 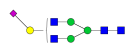 | H4N4E1 | 1820.66 | 1820.68 |
| 26 |  | H4N5F1 | 1850.69 | 1850.67 |
| 27 | 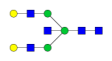 | H5N5 | 1866.67 | 1866.69 |
| 28 | 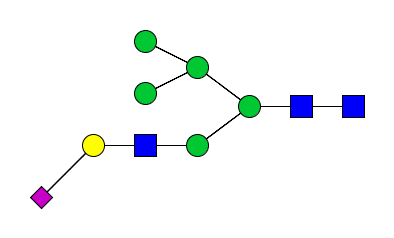 | H6N3L1 | 1895.64 | 1895.70 |
| 29 | 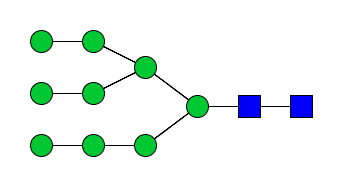 | H9N2 | 1905.63 | 1905.64 |
| 30 | 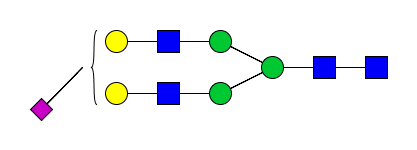 | H5N4L1 | 1936.67 | 1936.70 |
| 31 |  | H6N3E1 | 1941.68 | 1941.71 |
| 32 | 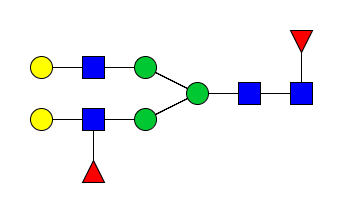 | H5N4F2 | 1955.70 | 1955.68 |
| 33 | 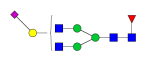 | H4N4F1E1 | 1966.71 | 1966.77 |
| 34 |  | H5N4E1 | 1982.71 | 1982.71 |
| 35 | 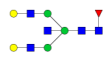 | H5N5F1 | 2012.72 | 2012.78 |
| 36 |  | H4N5E1 | 2023.73 | 2023.71 |
| 37 | 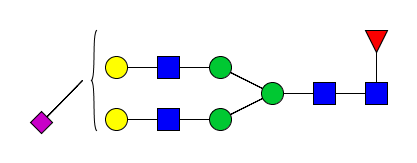 | H5N4F1L1 | 2082.72 | 2082.78 |
| 38 |  | H5N4F1E1 | 2128.77 | 2128.79 |
| 39 |  | H4N5F1E1 | 2169.79 | 2169.79 |
| 40 |  | H5N5E1 | 2185.79 | 2185.81 |
| 41 | 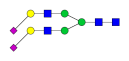 | H5N4L2 | 2209.75 | 2209.72 |
| 42 |  | H5N5E1Ac1 | 2227.80 | 2227.80 |
| 44 | 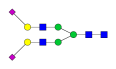 | H5N4L1E1 | 2255.80 | 2255.81 |
| 44 | 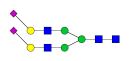 | H5N4E2 | 2301.83 | 2301.91 |
| 45 |  | H5N5F1E1 | 2331.84 | 2331.90 |
| 46 |  | H6N5E1 | 2347.84 | 2347.86 |
| 47 | 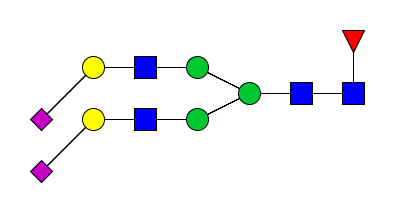 | H5N4F1L2 | 2355.81 | 2355.84 |
| 48 |  | H5N4F1L1E1 | 2401.85 | 2401.82 |
| 49 | 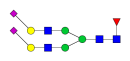 | H5N4F1E2 | 2447.89 | 2447.88 |
| 50 | 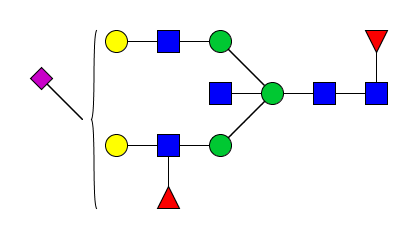 | H5N5F2E1 | 2477.90 | 2477.90 |
| 51 | 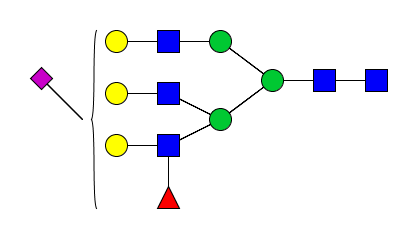 | H6N5F1E1 | 2493.90 | 2493.87 |
| 52 | 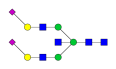 | H5N5E2 | 2504.91 | 2504.88 |
| 53 |  | H6N5L2 | 2574.88 | 2574.89 |
| 54 | 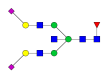 | H5N5F1L1E1 | 2604.93 | 2604.91 |
| 55 |  | H6N5L1E1 | 2620.93 | 2620.95 |
| 56 | 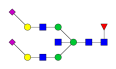 | H5N5F1E2 | 2650.97 | 2650.98 |
| 57 |  | H6N5E2 | 2666.98 | 2666.94 |
| 58 | 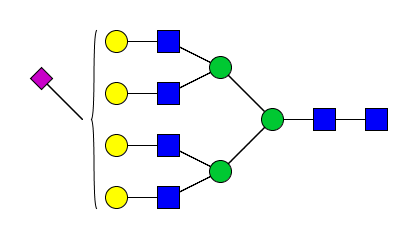 | H7N6E1 | 2712.97 | 2712.92 |
| 59 | 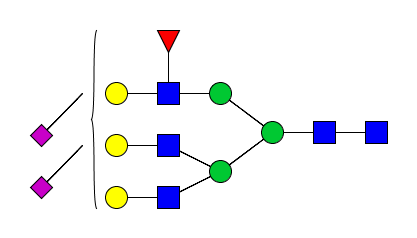 | H6N5F1L2 | 2720.94 | 2720.98 |
| 60 |  | H6N5F1L1E1 | 2766.98 | 2766.96 |
| 61 |  | H6N5F1E2 | 2813.02 | 2813.03 |
| 62 | 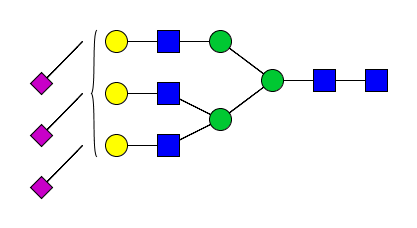 | H6N5L3 | 2847.97 | 2847.99 |
| 63 |  | H6N5L2E1 | 2894.01 | 2894.05 |
| 64 |  | H6N5L1E2 | 2940.05 | 2940.03 |
| 65 | 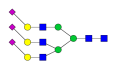 | H6N5E3 | 2986.09 | 2986.10 |
| 66 | 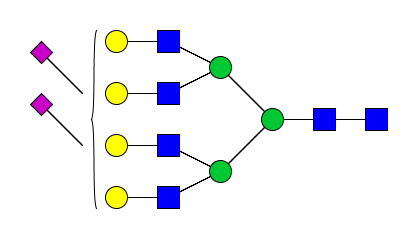 | H7N6E2 | 3032.10 | 3032.08 |
| 67 | 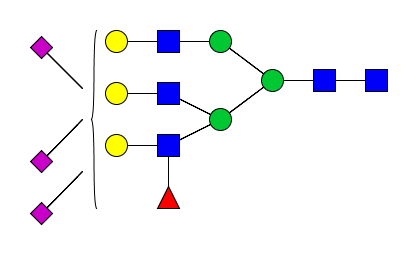 | H6N5F1L2E1 | 3040.07 | 3040.06 |
| 68 |  | H5N8L1E1 | 3068.16 | 3068.11 |
| 69 |  | H6N5F1L1E2 | 3086.10 | 3086.12 |
| 70 | 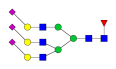 | H6N5F1E3 | 3132.15 | 3132.11 |
| 71 |  | H6N5F2L2E1 | 3186.13 | 3186.15 |
| 72 |  | H7N6L2E1 | 3259.14 | 3259.11 |
| 73 |  | H7N6L1E2 | 3305.18 | 3305.18 |
| 74 | 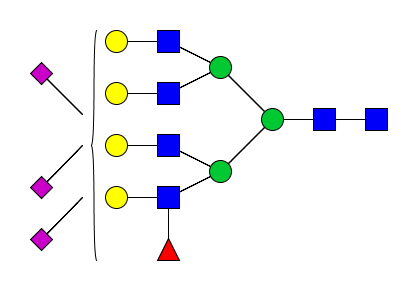 | H7N6F1L3E1 | 3405.20 | 3405.20 |
| 75 |  | H7N6L3E1 | 3532.23 | 3532.21 |
| 76 |  | H7N6L2E2 | 3578.27 | 3578.27 |
| 77 |  | H7N6L1E3 | 3624.31 | 3624.25 |
| 78 |  | H7N6F1L2E2 | 3724.40 | 3724.36 |

H = hexose, N = N-acetylhexosamine, F= fucose, L = lactonized N-acetylneuraminic acid (α2,3-linked), E = ethyl esterified N-acetylneuraminic acid (α2,6-linked), the number of residues given listed the letter. Green circle, Man; yellow circle, Gal; blue square, GlcNAc; red triangle, Fuc; clockwise purple diamond, α (2,6) -linked sialic acid; anticlockwise purple diamond, α (2,3) -linked sialic acid. The N-glycans with the CVs less than 25% according to the quantitation results are labeled with red.

**Supplemental Table 2. Different** **glyco-subclasses.**

| **Different glyco-subclasses** | |
| --- | --- |
| **Glyco-subclasses** | **Calculated** **formulas** |
| **Terminal-galactosylated N-glycans^1^ (TG)** | SUM[0.5×(H4N4+H4N4F1+H4N5F1)+1×(H4N3+H4N3F1+H5N4+H5N4F1+H5N5+H5N5F1)] |
| **Fucosylated N-glycans (F)** | SUM[H3N3F1+H4N3F1+H3N4F1+H4N4F1+H3N5F1+H4N3F1E1+H5N4F1+H4N5F1+H4N4F1E1+H5N5F1+H5N4F1L1+H5N4F1E1+H5N5F1E1+H5N4F1L2+H5N4F1L1E1+H5N4F1E2+H6N5F1E1+H5N5F1L1E1+H5N5F1E2+H6N5F1L2+H6N5F1L1E1+H6N5F1E2+H6N5F1L2E1+H6N5F1L1E2] |
| **High mannose N-glycans (M)** | SUM[H5N2+H6N2+H7N2+H8N2+H9N2] |
| **Hybrid N-glycans (Hy)** | SUM[H5N3+H5N3E1+H6N3L1+H6N3E1] |
| **α2,3-sialylated N-glycans^2^ (L)** | SUM[0.33×(H6N5L1E1+H6N5F1L1E1+H6N5L1E2+H6N5F1L1E2)+0.5×(H5N4L1+H5N4F1L1+H5N4L1E1+H5N4F1L1E1+H5N5F1LIE1+H5N8L1E1+H7N6L2E1)+0.67×(H6N5L2+H6N5F1L2+H6N5L2E1+H6N5F1L2E1)+1×(H4N3L1+H6N3L1+H5N4L2+H5N4F1L2+H6N5L3)] |
| **α2,6-sialylated N-glycans^3^ (E)** | SUM[0.25×(H7N6E1+H7N6F1L2E1)+0.33×(H6N5E1+H6N5F1E1+H6N5F1L1E1+H6N5F1L1E1+H6N5L2E1+H6N5F1L2E1)+0.5×(H5N4E1+H5N4F1E1+H5N5E1+H5N5E1Ac1+H5N4L1E1+H5N5F1E1+H5N4F1L1E1+H5N5F1L1E1+H5N8L1E1)+0.67×(H6N5E2+H6N5F1E2+H6N5L1E2+H6N5F1L1E2)+1×(H3N3E1+H4N3E1+H4N3F1E1+H5N3E1+H4N4E1+H6N3E1+H4N4F1E1+H5N4E2+H5N4F1E2+H5N4F1E2+H5N5E2+H5N5F1E2+H6N5E3)] |
| **Bisecting N-glycans (B)** | SUM[H3N5F1+H4N5F1+H5N5+H5N5F1+H5N5E1+H5N5E1Ac1+H5N5F1E1+H5N5F1L1E1+H5N5F1E2] |
| **Multi-branched Sialylated N-glycans^4^ (A2S,A3S)** | SUM[0.25×H7N6E1+0.33×(H6N5E1+H6N5F1E1)+0.67×(H6N5L2+H6N5L1E1+H6N5E2+H6N5F1L2+H6N5F1L1E1+H6N5F1E2)+0.75×H7N6L2E1+1×(H6N5L3+H6N5L2E1+H6N5L1E2+H6N5E3+H6N5F1L2E1+H6N5F1L1E2)] |

note:

**^1^** 0.50 to the structures: 1 Gal linked to 2 GlcNAc

1 to the structures: fully galactosylated glycans

**^2^** 0.33 to the structures: 1 L-NeuAc linked to 3 Gal

0.50 to the structures: 1 L- NeuAc linked to 2 Gal, or 2 L-NeuAc linked to 4 Gal

0.67 to the structures: 2 L-NeuAc linked to 3 Gal

1 to the structures: 1 L-NeuAc linked to 1 Gal, or 2 L-NeuAc linked to 2 Gal, or 3 L-NeuAc linked to 3 Gal

**^3^** 0.25 to the structures: 1 E-NeuAc linked to 4 Gal

0.33 to the structures: 1 E-NeuAc linked to 3 Gal

0.50 to the structures: 1 E- NeuAc linked to 2 Gal, or 2 E-NeuAc linked to 4 Gal

0.67 to the structures: 2 E-NeuAc linked to 3 Gal

1 to the structures: 1 E-NeuAc linked to 1 Gal, or 2 E-NeuAc linked to 2 Gal, or 3 E-NeuAc linked to 3 Gal

**^4^** 0.25 to the structures: 1 NeuAc linked to 4 Gal

0.33 to the structures: 1 NeuAc linked to 3 Gal

0.67 to the structures: 2 NeuAc linked to 3 Gal

0.75 to the structures: 3 NeuAc linked to 4 Gal

1 to the structures: 1 NeuAc linked to 1 Gal, or 2 NeuAc linked to 2 Gal, or 3 NeuAc linked to 3 Gal, or 4 NeuAc linked to 4 Gal

**Supplemental Table 3.**The relative quantitation results of 60 N-glycans between non-malignant and neuroblastoma serum.

| **No** | **Putative structure** | **m/z** | **Composition** | **Relative peak area**  **(Mean ± SD)** | | **Non-malignant vs** **Neuroblastoma** | |
| --- | --- | --- | --- | --- | --- | --- | --- |
|  |  |  |  | **Non-malignant** | **Neuroblastoma** | **p-value** | **AUC** |
| 1 | 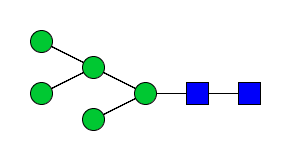 | 1257.46 | H5N2 | 0.0115±0.0034 | 0.0081±0.0029 | 3.0352E-5 | 0.7383 |
| 2 | 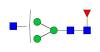 | 1282.44 | H3N3F1 | 0.0047±0.0022 | 0.0039±0.0025 | 0.1426 |  |
| 3 | 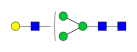 | 1298.46 | H4N3 | 0.0088±0.0045 | 0.0081±0.0055 | 0.5723 |  |
| 4 | 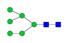 | 1419.49 | H6N2 | 0.0123±0.0042 | 0.0089±0.0041 | 0.0008 | 0.7439 |
| 5 | 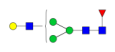 | 1444.54 | H4N3F1 | 0.0045±0.0021 | 0.0035±0.0023 | 0.0659 |  |
| 6 |  | 1455.52 | H3N3E1 | 0.0037±0.0014 | 0.0034±0.0015 | 0.3593 |  |
| 7 | 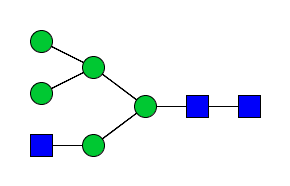 | 1460.48 | H5N3 | 0.0011±0.0004 | 0.0010±0.0005 | 0.4083 |  |
| 8 | 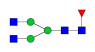 | 1485.54 | H3N4F1 | 0.0606±0.0235 | 0.0656±0.0364 | 0.5018 |  |
| 9 | 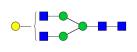 | 1501.57 | H4N4 | 0.0043±0.0013 | 0.0042±0.0023 | 0.8191 |  |
| 10 |  | 1571.58 | H4N3L1 | 0.0013±0.0005 | 0.0015±0.0010 | 0.3625 |  |
| 11 | 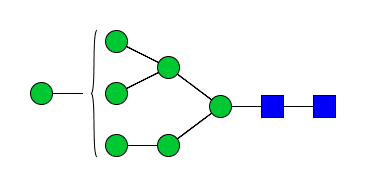 | 1581.51 | H7N2 | 0.0026±0.0010 | 0.0019±0.0008 | 0.0027 |  |
| 12 | 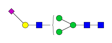 | 1617.58 | H4N3E1 | 0.0328±0.0157 | 0.0291±0.0129 | 0.2736 |  |
| 13 | 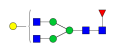 | 1647.65 | H4N4F1 | 0.0803±0.0296 | 0.0667±0.0332 | 0.0687 |  |
| 14 | 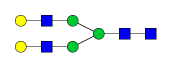 | 1663.59 | H5N4 | 0.0093±0.0071 | 0.0104±0.0083 | 0.5332 |  |
| 15 |  | 1688.65 | H3N5F1 | 0.0042±0.0014 | 0.0055±0.0048 | 0.1400 |  |
| 16 |  | 1743.61 | H8N2 | 0.0035±0.0014 | 0.0025±0.0012 | 0.0035 |  |
| 17 | 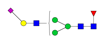 | 1763.66 | H4N3F1E1 | 0.0026±0.0011 | 0.0031±0.0035 | 0.4686 |  |
| 18 |  | 1779.68 | H5N3E1 | 0.0033±0.0010 | 0.0029±0.0012 | 0.1207 |  |
| 19 | 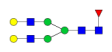 | 1809.67 | H5N4F1 | 0.0356±0.0117 | 0.0267±0.0166 | 0.0114 |  |
| 20 | 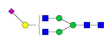 | 1820.68 | H4N4E1 | 0.0041±0.0014 | 0.0032±0.0014 | 0.0073 |  |
| 21 |  | 1850.67 | H4N5F1 | 0.0047±0.0012 | 0.0049±0.0031 | 0.7504 |  |
| 22 | 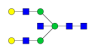 | 1866.69 | H5N5 | 0.0011±0.0005 | 0.0012±0.0007 | 0.6019 |  |
| 23 | 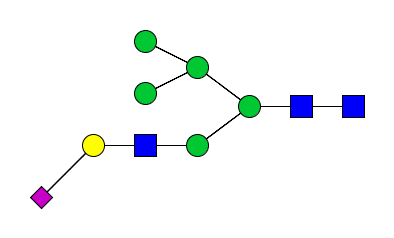 | 1895.70 | H6N3L1 | 0.0008±0.0003 | 0.0008±0.0007 | 0.6164 |  |
| 24 | 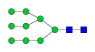 | 1905.64 | H9N2 | 0.0048±0.0016 | 0.0035±0.0013 | 0.0002 | 0.7545 |
| 25 | 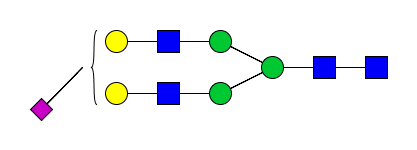 | 1936.70 | H5N4L1 | 0.0026±0.0009 | 0.0026±0.0014 | 0.8633 |  |
| 26 |  | 1941.71 | H6N3E1 | 0.0023±0.0007 | 0.0019±0.0009 | 0.0199 |  |
| 27 | 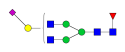 | 1966.77 | H4N4F1E1 | 0.0030±0.0009 | 0.0025±0.0011 | 0.0280 |  |
| 28 |  | 1982.71 | H5N4E1 | 0.0852±0.0191 | 0.0764±0.0252 | 0.0966 |  |
| 29 | 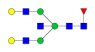 | 2012.78 | H5N5F1 | 0.0017±0.0005 | 0.0013±0.0005 | 0.0044 |  |
| 30 | 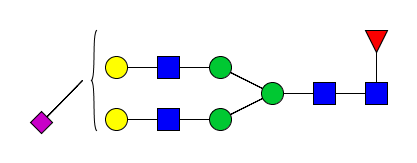 | 2082.78 | H5N4F1L1 | 0.0056±0.0024 | 0.0046±0.0027 | 0.1070 |  |
| 31 |  | 2128.79 | H5N4F1E1 | 0.0262±0.0076 | 0.0190±0.0083 | 0.0002 | 0.7364 |
| 32 |  | 2185.81 | H5N5E1 | 0.0027±0.0012 | 0.0025±0.0016 | 0.5757 |  |
| 33 | 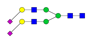 | 2209.72 | H5N4L2 | 0.0015±0.0003 | 0.0015±0.0005 | 0.6432 |  |
| 34 |  | 2227.80 | H5N5E1Ac1 | 0.0008±0.0002 | 0.0009±0.0004 | 0.0748 |  |
| 35 | 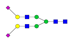 | 2255.81 | H5N4L1E1 | 0.0359±0.0116 | 0.0311±0.0117 | 0.0843 |  |
| 36 | 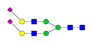 | 2301.91 | H5N4E2 | 0.3501±0.0384 | 0.3612±0.0663 | 0.3998 |  |
| 37 |  | 2331.90 | H5N5F1E1 | 0.0092±0.0047 | 0.0073±0.0044 | 0.0977 |  |
| 38 |  | 2347.86 | H6N5E1 | 0.0059±0.0023 | 0.0063±0.0023 | 0.4800 |  |
| 39 | 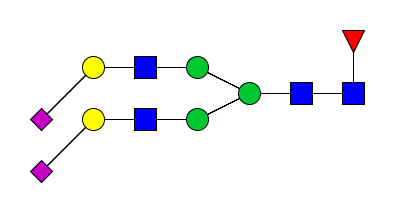 | 2355.84 | H5N4F1L2 | 0.0036±0.0013 | 0.0049±0.0031 | 0.0272 |  |
| 40 |  | 2401.82 | H5N4F1L1E1 | 0.0122±0.0048 | 0.0207±0.0129 | 0.0010 |  |
| 41 | 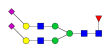 | 2447.88 | H5N4F1L1E1 | 0.0267±0.0105 | 0.0278±0.0117 | 0.6720 |  |
| 42 | 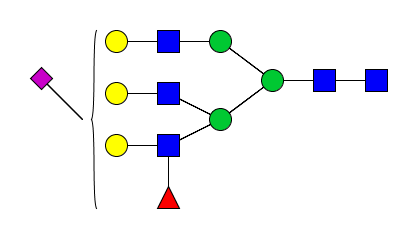 | 2493.87 | H6N5F1E1 | 0.0011±0.0003 | 0.0022±0.0005 | 0.1916 |  |
| 43 | 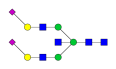 | 2504.9 | H5N5E2 | 0.0015±0.0005 | 0.0023±0.0018 | 0.0126 |  |
| 44 |  | 2574.89 | H6N5L2 | 0.0009±0.0003 | 0.0020±0.0029 | 0.0356 |  |
| 45 | 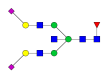 | 2604.91 | H5N5F1L1E1 | 0.0013±0.0004 | 0.0016±0.0007 | 0.0192 |  |
| 46 |  | 2620.95 | H6N5L1E1 | 0.0117±0.0049 | 0.0139±0.0072 | 0.1537 |  |
| 47 | 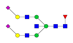 | 2650.98 | H5N5F1E2 | 0.0055±0.0037 | 0.0054±0.0045 | 0.9336 |  |
| 48 |  | 2666.94 | H6N5E2 | 0.0060±0.0022 | 0.0072±0.0032 | 0.0590 |  |
| 49 | 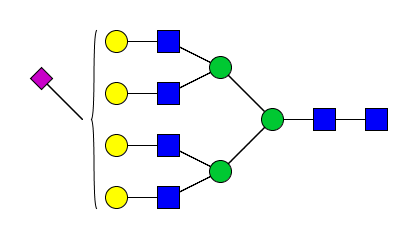 | 2712.92 | H7N6E1 | 0.0004±0.0001 | 0.0005±0.0002 | 0.1133 |  |
| 50 | 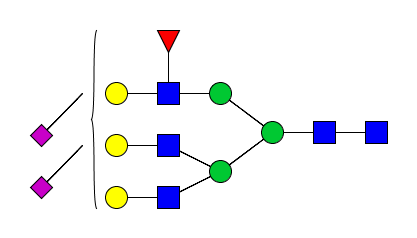 | 2720.98 | H6N5F1L1E1 | 0.0024±0.0009 | 0.0029±0.0019 | 0.1770 |  |
| 51 |  | 2766.96 | H6N5F1L1E1 | 0.0022±0.0011 | 0.0045±0.0034 | 0.0008 | 0.7735 |
| 52 |  | 2813.03 | H6N5F1E2 | 0.0006±0.0002 | 0.0008±0.0004 | 0.0094 |  |
| 53 | 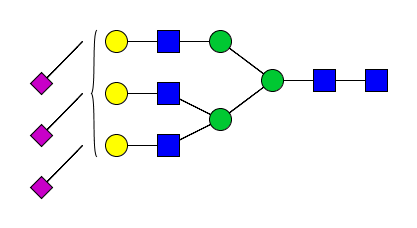 | 2847.99 | H6N5L3 | 0.0004±0.0001 | 0.0005±0.0002 | 0.0277 |  |
| 54 |  | 2894.05 | H6N5L2E1 | 0.0045±0.0018 | 0.0050±0.0032 | 0.4465 |  |
| 55 |  | 2940.03 | H6N5L1E2 | 0.0461±0.0128 | 0.0477±0.0211 | 0.6984 |  |
| 56 | 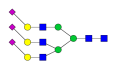 | 2986.09 | H6N5E3 | 0.0151±0.0074 | 0.0220±0.0141 | 0.0145 |  |
| 57 | 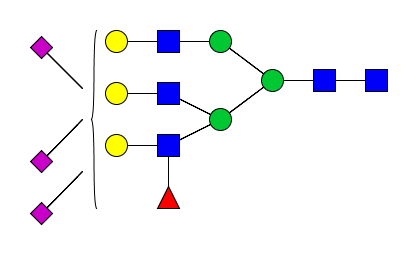 | 3040.05 | H6N5F1L2E1 | 0.0009±0.0004 | 0.0017±0.0013 | 0.0015 |  |
| 58 |  | 3068.11 | H5N8L1E1 | 0.0004±0.0002 | 0.0009±0.0008 | 0.0029 |  |
| 59 |  | 3086.12 | H6N5F1L1E2 | 0.0110±0.0075 | 0.0241±0.0193 | 0.0007 | 0.7409 |
| 60 |  | 3259.11 | H7N6L2E1 | 0.0005±0.0002 | 0.0005±0.0003 | 0.4454 |  |

H = hexose, N = N-acetylhexosamine, F= fucose, L = lactonized N-acetylneuraminic acid (α2,3-linked), E = ethyl esterified N-acetylneuraminic acid (α2,6-linked), the number of residues given listed the letter. Green circle, Man; yellow circle, Gal; blue square, GlcNAc; red triangle, Fuc; clockwise purple diamond, α (2,6) -linked sialic acid; anticlockwise purple diamond, α (2,3) -linked sialic acid.


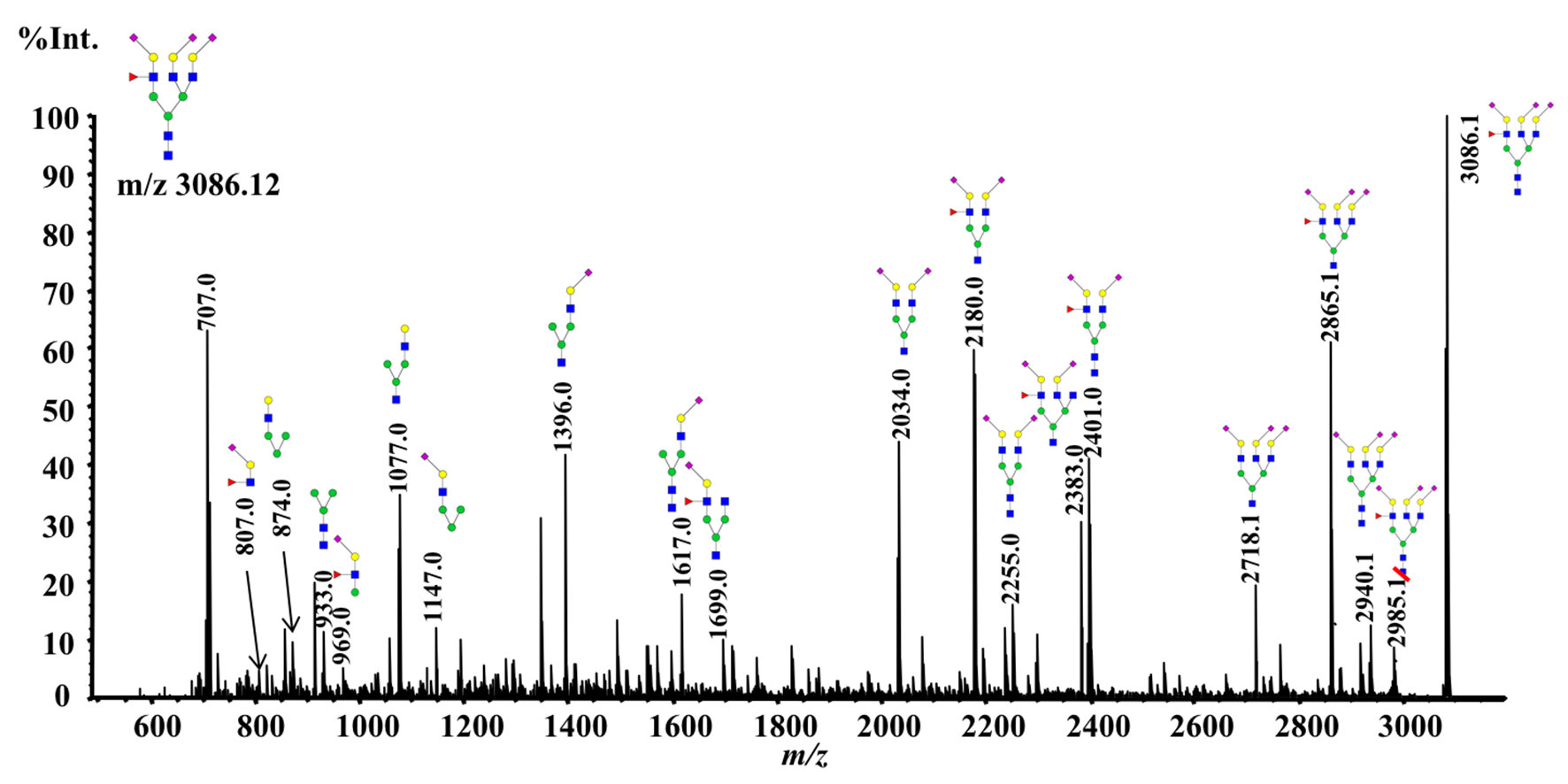


**Supplementary Figure 1:** MALDI-QIT-TOF tandem mass spectra of precursor ion [H6N5F1L1E2 + Na]^+^ (m/z 3086.12) in positive ion mode. The red line indicates an 0,2A crossing fragment. Green circle, Man; yellow circle, Gal; blue square, GlcNAc; clockwise purple diamond, α (2,6) -linked sialic acid; anti-clockwise purple diamond, α (2,3) -linked sialic acid.
